# Wild Felids Blood Group System

**DOI:** 10.3390/ani11123533

**Published:** 2021-12-11

**Authors:** Ana Silvestre-Ferreira, Josep Pastor

**Affiliations:** 1 Department of Veterinary Sciences, University of Trás-os-Montes and Alto Douro (UTAD), Quinta de Prados, 5000-801 Vila Real, Portugal; 2 Animal and Veterinary Research Centre (CECAV), University of Trás-os-Montes and Alto Douro (UTAD), Quinta de Prados, 5000-801 Vila Real, Portugal; 3 Department de Medicinia i Cirurgia Animals, Facultat de Veterinària, Universititat Autònoma de Barcelona, Bellaterra, 08193 Barcelona, Spain; Josep.Pastor@uab.cat

**Keywords:** wild felids, blood type, cat, alloantibody, blood transfusion

## Abstract

**Simple Summary:**

The AB blood group system has been identified in wild felids, as well as in the domestic cat. In both, type A blood seems to be the most common, although the majority of wild felid species exhibit one single blood type, showing that there seems to be variation between species, but not within species, and no evidence of geographical variation was yet found, showing apparently no genetic variability. Further studies are necessary to determine the clinical relevance of the AB blood group on wild felids. This manuscript makes a complete and comprehensive review of the knowledge on wild felids blood groups and transfusion medicine.

**Abstract:**

Wild felids and domestic cats share the AB blood group. However, there have been few studies regarding the characterization and prevalence of the different blood types in wild animals. The erythrocyte membrane glycolipids of the wild cats correspond to the major disialoganglioside patterns observed in domestic cats. Like in domestic cats, type A blood seems to be the most common, although wild felid species seem to exhibit one single blood type. Of the species studied, the wild domestic cats, and the Panthera and ocelot lineages, all had type A blood; the Puma lineage showed almost exclusively type B blood. The prevalence of wild felids blood types show that there seems to be variation between species, but not within species, and no evidence of geographical variation has yet been found, showing apparently no genetic variability. The presence of alloantibodies has also been demonstrated, so the risk of life-threatening transfusion reactions due to mismatched transfusions and neonatal isoerythrolysis is a possibility. Like in other species, the recognition of wild felids blood groups is clinically relevant, as it can also be important in establishing phylogenetic relationships within the Felidae family. We will review the current knowledge on this topic and give insights into the wild felids blood groups potential for zoo transfusion medicine and phylogenetic studies in order to help support reintroduction projects and to preserve genetic diversity.

## 1. Introduction

Knowledge of blood type systems in animals is of paramount importance mainly for blood transfusion medicine, phylogenetic studies, and reproductive compatibilities. Concerning phylogenetic studies, the analysis of the phylogenetic tree for the primate ABO blood group allowed for an estimation of the divergence time between human and nonhuman primates [1]. Although sharing a similar nomenclature, the human ABO blood group and the feline AB blood group do not share the same glycolipid antigenic determinants of the erythrocyte membrane [2]. Recently, in order to avoid misinterpretations, it was proposed that the feline blood group system should be renamed to ABC, where C corresponds to the blood type AB [3]. In felids, the divergence is estimated to have occurred over 9000 years ago, where the domestic cat was considered a member of the subspecies Felis silvestris catus, derived from the domestication of the Libyan cat (Felis silvestris lybica). Genetic studies show that, in some European regions, most European wildcats are hybrids between the wild and domestic subspecies [4]. However, the phylogenetic relationships among the felids need to be further studied, and it is also unknown how the feline blood type systems have been inhered among the Felidae family.

Blood transfusions can be life-saving when required [5]. In wild felids medicine, there are common problems like run overs, parasitism, blood loss, and hypotension associated with surgical procedures, where a blood transfusion can be a life-saving procedure [6,7]. Reports of blood transfusions in species other than dogs and cats are very scarce in the literature, even more so in regard to wild felids [6]. In order to perform a blood transfusion in a safe manner, there is a need for knowledge of feline blood groups; however, blood groups remain mostly unstudied or unknown for wild felids [5,6]. Mismatched transfusions can produce immune reactions that result in red blood cells hemolysis and recipient death; blood typing and crossmatching ensure compatible and effective transfusions [8,9]. Xenotransfusions have been tried, both between domestic cats and wild cats, or even between dogs and domestic cats, but the results do not always lead to an effective result [10,11,12,13,14].

Following the above considerations, it is of paramount importance to recognize the blood groups in wild felids. In the present article, we review the state of the art of wild felids blood group system and future research needs.

## 2. AB Blood System in Domestic Cats

In domestic cats, the AB blood system is the most important and well-studied blood group system. Cats are classified as types A, B, or AB based on the molecular nature of the blood group antigen gangliosides [2,15,16]. The glycolipid antigenic determinants of the erythrocyte membrane are N-acetylneuraminic acid (NeuAc) and N-glycolylneuraminic acid (NeuGc). Blood types are determined by mutations in cytidine monophospho-N-acetylneuraminic acid hydroxylase (CMAH), the enzyme responsible for the conversion of NeuAc to NeuGc [17]. According to a recent study, all cats with type B and AB carry two alleles derived from the mutated CMAH gene highly associated with the expression of the NeuAc [18]. Type B erythrocytes possess only NeuAc acid, type A erythrocytes possess primarily NeuGc, but NeuAc can also be present, while type AB cats co-express NeuAc and NeuGc, as well as intermediate forms of gangliosides [2,15,16]. There are no reports of cats lacking both A and B red cell antigens [19]. Type A and type B are inherited as a simple autosomal mendelian trait, with A being dominant over B [20]. Type A blood cats may have AA, or Ab genotype. Type B cats are always homozygote. AB is allelic to A and B and is represented as A > aab > b. Possible genotypes/phenotypes would be AA (Type A), Aaab (Type A), Ab (Type A), aab b (Type AB) and aab aab (Type AB), and bb (Type B) [17]. In general, type A blood type is the most common, type B is less common, and type AB is rare, with a global frequency lower than 1%. These frequencies vary geographically between and within countries, with type A being the most common blood type in non-pedigree cat populations in different countries, as reported by several studies worldwide [2,21,22,23,24,25,26,27,28,29,30,31,32,33,34,35,36,37,38,39,40,41,42,43,44,45,46,47]. Variations are also described among breeds, with type B showing a high frequency in some pure breeds [21,48,49,50]. A new erythrocyte antigen, the Mik antigen, has been described, but its worldwide prevalence is still unknown [51].

Domestic cats can possess naturally occurring alloantibodies to the blood type they lack. Naturally occurring alloantibodies are directed against the glycolipid antigenic determinants of the erythrocyte membrane. All type B adult cats have high titers of naturally occurring anti-A alloantibodies, but only approximately one-third of type A adult cats have naturally occurring anti-B alloantibodies [19,52]. No alloantibodies have been found in type AB cats [8,24,28,46,53,54]. Alloantibodies are responsible for hemolytic transfusion reactions in mismatched blood transfusions [9,55] that may vary from premature erythrocyte destruction to acute, severe, and potentially fatal recipient reactions [8,56,57]. The degree of the reaction is proportional to the antibody titer of the recipient animal [54]. Neonatal isoerythrolysis may occur in type A or AB kittens born to a type B queen because of the anti-A antibodies present in colostrum [58,59,60]. Although the alloantibodies are lacking in type-AB cats, animals may present transfusion reactions when receiving blood types other than AB, due to anti-A or anti-B alloantibodies in the donor plasma [55]. It is thought that antibodies to these determinants are present innately, presumably originating from environmental or dietary exposure to gastrointestinal bacteria, or plant epitopes, which have structural components that are similar to erythrocyte blood group antigens [8]. 

## 3. Wild Felids AB Blood System

**Figure 1:**
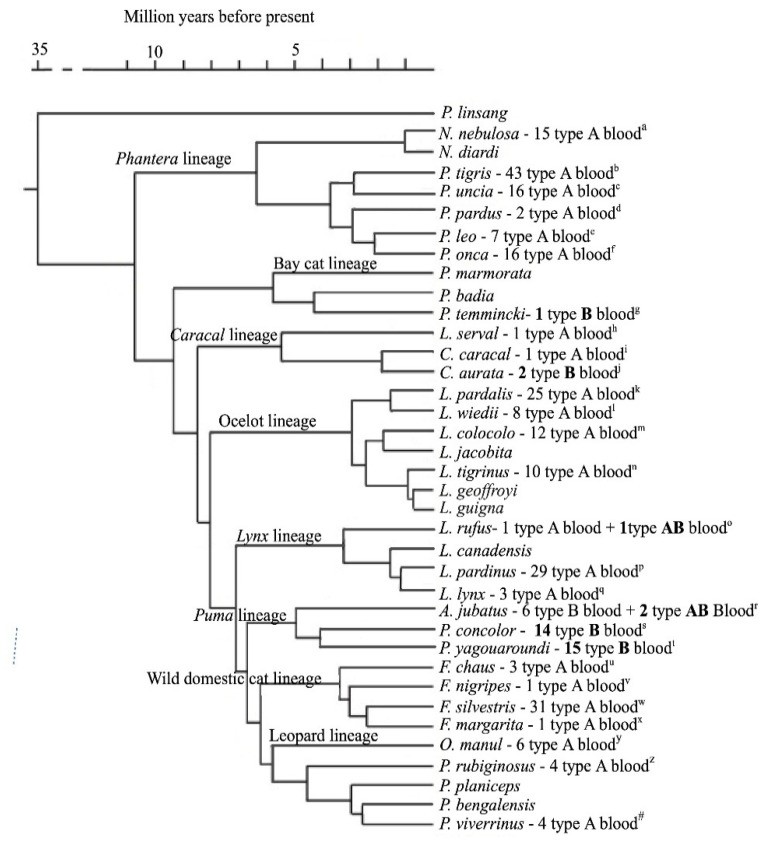
Phylogeny of Felidae, with the number of animals studied, their blood type, and place of origin. Adapted from Werdelin (2010) [62]. ^a,c,d,e,g,h,i,j,o,q,r,u,v,x,y,z,#^ USA/Dubai [61]; ^b^ 30 Thailand [6] + 13 USA/Dubai [61]; ^f,l,s^ 5 USA/Dubai [61] + 11 Brazil [65]; ^k,t^ 9 USA/Dubai [61] + 8 Brazil [7] + 8 Brazil [65]; ^m^ 5 USA/Dubai [61] + 7 Brazil [7]; ^n^ Brazil [65]; ^p^ Spain—subsequently expanded to 140 animals [64]; ^w^ 6 USA/Dubai [61] + 25 Spain [63]. In bold, species where type B or type AB blood were found.

A study on 131 wild felids of 26 different species from zoos and wild animal parks in the USA and Dubai identified for the first time the AB blood group system in the species studied [61]. The authors also demonstrated, by high performance thin layer chromatography, that erythrocyte membrane glycolipids of the wild cats correspond to the major disialoganglioside patterns observed in domestic cats, with type B cells expressing exclusively NeuAc and type A cells predominantly NeuGc acid, with small amounts of NeuAc [2,15,16,61]; however, studies on CMAH mutations are lacking. Thus, it has been assumed that the traditional and the commercial feline blood typing methods could also be applied for wild felids blood typing [60]. Since then, a few new studies on wild felids blood group system have been performed in different species and with different blood typing methods (Table 1). In Figure 1, according to a study on phylogeny of Felidae based on a data set of 22,789 base pairs of DNA, including autosomal, Y-linked, X-linked, and mitochondrial gene segments [62], we added to the phylogenetic tree data showing the number of animals studied, their blood type and place of origin. Naturally occurring alloantibodies were also studied [61,63,64] and identified [63,64].

Type A blood seems to be the most common among wild species as it is in domestic cats [21,22,23,24,25,26,27,28,29,30,31,32,33,34,35,36,37,38,39,40,41,42,43,44,45,46,47] (Table 1 and Figure 1). All animals belonging to the wild domestic cats lineage presented type A blood. Regarding the *Lynx, Panthera*, and ocelot lineages, all animals presented type A blood, but one animal (*Lynx rufus*) [61] exhibited type AB blood. Almost every cat in the *Puma* lineage presented blood type B, with the exception of two cheetahs (*Acinonyx jubatus*) [61] presenting blood type AB. Type B blood was also identified in African (*Caracal aurata*) and Asian golden cats (*Pardofelis temminckii*). The phylogenetic relationship between these species is not yet completely understood. In domestic cats, no breed was identified exclusively to have type B blood; however, breeds such as British shorthaired [24] as well as Turkish Van and Angora [48] have a prevalence of animals with type B blood of 58.7%, 60%, and 46.4%, respectively. Three animals were described with AB blood type, two cheetahs (*Acinonyx jubatus*) and a brown lynx (*Lynx rufus*), raising the hypothesis that this blood type is easier to find in African and Asian species [61]. No other AB blood type animal was found in the other studies performed. AB blood type animals were found in a population of only type B blood animals for cheetahs, and only type A blood for lynxes, although only eight cheetahs and four lynxes were evaluated. No B blood type was found in all of the 140 lynxes (*Lynx pardinus*) evaluated in Spain [64]. No other study evaluated cheetahs.

The overall presentation of wild felids blood types show that there is variation among breeds, but not within breeds, and no evidence of geographical variation has yet been found, showing apparently no genetic variability and probably reflecting a relatively minor gene pool within wild felid species in contrast to domestic cats. In domestic cats, according to a recent study, there is no evidence of the relationship between blood types and phylogenetic origin, despite some similarities found in blood types prevalences between Asian and American breeds and European and Oceanian breeds that could be explained by geographical proximity and trade relations in the first case and colonization policies in the second [66]. Recently, five feline erythrocyte antigens were identified [67], raising the suspicion that other blood group erythrocyte antigens might exist either for domestic cats or wild felids. Genetic and molecular studies in a larger number of animals are needed in order to unravel the presence of new blood groups.

The absence of naturally occurring alloantibodies was reported in all species evaluated in USA and Dubai [61]. Subsequent studies in Europe revealed the presence of alloantibodies in lynxes (*Lynx pardinus*) and wild domestic cats (*Felis silvestris*) [63,64]. Three out of 26 (11.5%) lynxes [64] and four out of 25 (16%) of the wildcats [63] were found to have anti-B antibody titers. Furthermore, in wild cats, the pedigree study of 22 related animals showed that the cats with anti-B antibodies had the same ancestor [63]. The presence of anti-B alloantibodies among wild felids seems to be similar to the domestic cats, where about one-third of type A blood cats present anti-B alloantibodies [8,19,28,52,53,54]. On the other hand, incompatible blood crossmatches done across members of different phylogenetic groups indicate the possibility of felid group-specific erythrocyte antigens [63]. Identical results were found in Thailand [6]: when performing several crossmatch tests between tiger and domestic cat blood, the authors observed the reaction of tiger plasma with type A blood from cats, suggesting the possibility of another blood type in tigers. Recently, similar results have been found in domestic cats, where after performing 1228 crossmatches with blood from 258 type A cats, seven new naturally occurring alloantibodies outside the AB blood group system were identified [67]. Nevertheless, 80% of major and 60% of minor compatibilities were found between blood from 10 tigers and 10 domestic cats with blood type A [6]. In Brazil, crossmatch tests performed between haystack (*Leopardus colocolo*) and domestic cats were compatible, although some demonstrated the presence of rouleaux, which suggests that blood transfusions between these species can be safe [7]. Still, this same work demonstrated that the blood of the type B domestic cat cannot be used for gray cat (*Puma yagouaroundi*) [7]. A recent study on xenotransfusion of canine packed red blood cells to cats revealed this practice to be possible, but hemolysis should be expected between one and six days after transfusion [14]. The benefits and absence of severe adverse effect observed by researchers [6,7,10,11,12,13,14], as well as the relative ease of collecting blood from domestic cats, may support the relative safety of xenotransfusion from domestic to wild cats and, therefore, its application in emergency case. Further studies are needed to evaluate the behavior of allogeneic blood in vivo.

In domestic cats, blood loss is the most common indication for blood transfusion, followed by ineffective erythropoiesis and red blood cells destruction [68]. Little is known about the incidence of such diseases in wild felids. Blood loss in wild felids may be related to run overs, surgical procedures, or parasitism [6,7]. A study in Mato Grosso, Brazil, describes that from 211 run overs, 59. were mammals, mostly represented by carnivores, with 52 roadkilled individuals (24% of all roadkilled animals), including some rare or endangered species such as *Puma yagouaroundi, Leopardus colocolo, Puma concolor*, and ocelot (*Leopardus pardalis*) [69], but no reports of blood transfusion were found. As far as we know, no erythropoiesis disorders have been described in wild felids, but there has been a report of red blood cells destruction related to a rattlesnake bite in a caracal [70]. As a major cause of blood transfusion related to hemolytic anemia in domestic cats, erythrocyte parasitism, like hemoplasmosis, babesiosis, or cytauxzoonosis, has also been reported in wild felids [71,72,73,74,75,76,77], and is sometimes related to anemia [71,73,76], but there have been no reports of animals receiving blood transfusions. As in domestic cats, in wild felids, to ensure compatible transfusions, blood typing and crossmatching should be performed. As mismatched transfusions can produce immune reactions that might result in red blood cells hemolysis and recipient death [8,9], a major and minor crossmatch prior to blood transfusion should always be performed and, whenever possible, a blood group determination should be conducted [78,79,80]. However, the clinical relevancy of the AB blood group system in wild felids is still not clear. The finding of only one blood type within the majority of wild felids species evaluated can allow, in those species where blood groups have not been defined, reliance on a single transfusion that must always be preceded by a crossmatch. Sometimes, upon compatibility studies, the use of domestic cat blood can lead to a safe blood transfusion. However, because of the presence of naturally occurring alloantibodies, post transfusion reactions or neonatal isoerythrolysis cannot be completely ruled out.

As blood groups are not well defined in wild felids, leaning on crossmatching to ensure a safe blood transfusion may be an alternative to identify circulating blood group antibodies, however it cannot determine blood groups [5].

Although blood transfusions can be life-saving, in domestic cats and dogs blood transfusion medicine there are descriptions of risks associated with the transmission of infectious and transmissible parasitic diseases that should also be taken in account when performing blood transfusions in wild cats [81,82,83,84,85,86].

## 4. Conclusions

Unfortunately, the amount of research conducted on this topic is scarce, however, although an absence of genetic variability has been observed, as wild felid species seem to exhibit one single blood type, the information presented here and the recent knowledge on domestic cat blood types leads us to suspect the possibility that blood groups other than AB exist in wild felids. Particular attention should be paid to the presence of naturally occurring alloantibodies. In endangered wild felids in captivity, common problems in clinical and surgical management might include blood transfusions for its resolution; however, the number of blood donors is extremely limited and are generally not available. In the case of an urgent need for a blood transfusion, upon a crossmatch test, domestic cat blood could be used, but more studies are required before attempting xenotransfusion among felid groups. Taking into account our data, wild felids blood types knowledge could be useful not only for zoo medicine, but also for phylogenetic studies for reintroduction projects in order to preserve genetic diversity. More studies, with more species and a greater number of animals, are needed in order to clarify the importance of the AB blood group system in wild felids and its clinical relevance. In addition, because the majority of the European wildcats are hybrids between wild and domestic subspecies, we are currently left to wonder what mutations occurred in the CMHA 9000 years ago when the divergence from the wild cat to the domestic cat took place.

## Figures and Tables

**Table 1:** Frequencies of blood type A, B, and AB in wild felids based on different countries, blood typing methods, and the presence of alloantibodies.

Lineage	Nº Animals	Blood Type			Typing Method	Alloantibodies	Country
		A	B	AB	-		
Ocelot	15	15			Tube hemagglutination	Not detected	USA/Dubai [61]
*Caracal*	8	6	2				
Asian leopard	4	4					
*Puma*	23		21	2			
*L* *ynx*	5	4		1			
Bay cat	1		1				
Wild domestic cats	17	17					
*Panthera*	58	58					
*Felis silvestris*	25	25			Tube hemagglutination	Detected	Spain [63]
*Lynx pardinus*	29	29			Tube hemagglutination	Detected	Spain [64]
*Lynx pardinus*	111	111			Immunochromatographic	Not determined	Spain *
*Panthera tigris tigris*	30	30			Slide agglutination test	Not determined	Thailand [6]
Ocelot	15	15			Tube hemagglutination	Not determined	Brazil ** [7]
*Puma*	8		8				
Ocelot	25	25			Tube hemagglutination and card ***	Not determined	Brazil **** [65]
*Puma*	6		6				
*Panthera*	11	11					

* Unpublished material from authors, blood typing with QuickTest BT A+B, Alvedia (Lyon, France); ** Goias State; *** RapidVet H Feline Blood typing, DMS laboratories (New Jersey, USA); **** States of São Paulo, Paraná, Botucatu, Roraima, Pará, Amazonas.

## Data Availability

Not applicable.
